# Analysis on the Spatial-Temporal Evolution Characteristics and Spatial Network Structure of Tourism Eco-Efficiency in the Yangtze River Delta Urban Agglomeration

**DOI:** 10.3390/ijerph18052577

**Published:** 2021-03-04

**Authors:** Yiyang Sun, Guolin Hou

**Affiliations:** 1 School of Geographic Science, Nanjing Normal University, Nanjing 210023, China; 191302038@njnu.edu.cn; 2 Jiangsu Center for Collaborative Innovation in Geographical Information Resource Development and Application, Nanjing 210023, China

**Keywords:** tourism eco-efficiency, spatial-temporal evolution, spatial network structure, Yangtze River Delta urban agglomeration

## Abstract

Based on the panel data of 41 cities in the Yangtze River Delta from 2008 to 2017, this paper constructs an evaluation indicators system for urban tourism eco-efficiency. By measuring the tourism eco-efficiency in the Yangtze River Delta urban agglomeration, we analyze its spatial-temporal evolution characteristics. Furthermore, the modified gravity model and social network analysis are introduced to explore the spatial network structure of tourism eco-efficiency and its evolution trend.The results show that:(1) The overall eco-efficiency of tourism in the Yangtze River Delta region presents a fluctuating downward trend, among which Jiangsu and Zhejiang have high eco-efficiency, Shanghai and Anhui are relatively low. The gap within the region first increased and then decreased. (2) During this decade, the spatial network structure of tourism eco-efficiency in the Yangtze River Delta has become increasingly loose. The weakening of the network connection strength has led to a decrease in the regional tourism eco-efficiency to a great extent. (3) The network centrality of cities such as Zhoushan, Huzhou, and Huangshan has always maintained a high level, and these cities have firmly occupied the core position of network. (4) The spatial association network of tourism eco-efficiency can be divided into four blocks: “two-way spillover”, “net spillover”, “net benefit” and “agent”. The synergy and spillover effect between various blocks are significant, and there is a spatial polarization trend centered on a few cities. Based on this, this paper puts forward optimization suggestions for the spatial network structure of the Yangtze River Delta urban agglomeration, in anticipation of promoting the improvement of regional tourism eco-efficiency.

## 1. Introduction

For a long time, tourism has been regarded as an environmentally friendly industry with low emissions, low energy consumption and low pollution. Besides, it has strong driving capacity of consumption and employment. Therefore, the active development of tourism has almost become the consensus of all governments. However, in recent years, with the rapid development of tourism industry and the continuous expansion of investment scale, the environmental impact caused by it has gradually been exposed. According to the research of the World Tourism Organization (UNWTO), the global carbon emissions of the tourism industry account for 5%–14% of the total carbon emissions from human activities. If not controlled, the global carbon emissions will increase at a rate of 2.5% per year [1]. Tourism activities consume a large amount of energy, resulting in air pollution, water pollution, solid waste pollution and carbon dioxide emission, all of which threaten the environmental quality of tourism destinations. Therefore, the concept of “sustainable tourism” was clearly put forward in the “Sustainable Tourism Development Action Strategy”, which was issued at the International Conference on Sustainable Development in 1990. At the same time, its objectives and main framework were pointed out. Sustainable tourism development refers to meeting the tourist demand of contemporary people without harming the possibility of tourism development for future generations to meet their tourist demand. It emphasizes that attention must be paid to the organic unity of economic, environmental, and social benefits while developing tourism [2]. However, with the global warming and the destruction of ecological environment, how to complete the green transformation of tourism industry and achieve its own sustainable development has become a common problem to be solved urgently in the world today. Therefore, research on the eco-efficiency of tourism industry has also emerged.

Eco-efficiency is defined as the efficiency of economic activities that takes into account resource utilization and environmental impact, it was first proposed by Schaltegger and Sturm to promote the sustainable development of the business sector [3]. In 1992, the World Business Council for Sustainable Development (WBCSD) further clarified the concept of eco-efficiency and promoted it in the business sector [4]. Later, ESCAP expanded its application scope to the economy-wide level, and explored developing eco-efficiency indicators to measure the status and progress of eco-efficiency in the economy, so as to provide decision-making departments with socio-economic policies related to environmental sustainability. Eco-efficiency focuses on achieving the progress of economy and environment through more efficient use of resources and lower pollution, and is considered as one of the useful tools for measuring green growth [5]. In 2005, Gössling introduced eco-efficiency into tourism research for the first time, and made a quantitative analysis of the economic value and environmental impact produced by tourism [6]. Tourism eco-efficiency is a key index to measure the degree of green development of tourism industry, its core idea is that tourism activities use the minimum environmental cost to create the greatest economic benefits. It has become an important strategic tool for evaluating the sustainable development of regional tourism [7].

As the most dynamic geographic unit in economic development pattern of a country, urban agglomerations are gradually becoming an important platform and leading force for regional economic and social development [8]. In urban agglomerations, different urban areas can be linked through spatial interactions in many aspects such as society, economy, energy, and carbon dioxide emissions [9]. Besides, due to geographical proximity, there are often a large number of complex and changeable tourist flows within urban agglomerations [10], and cities have relatively close ecological connections [11]. Therefore, tourism eco-efficiency of each city within the urban agglomeration not only depends on its own tourism development, but also is affected by other surrounding cities, and sometimes even breaks through the limitation of geographic space. Research shows that the tourism eco-efficiency has significant spatial association characteristics due to the superimposed influence of regional economic development level, tourism technology level, and industrial structure [12]. Meanwhile, the spatial structure of tourism eco-efficiency is increasingly diversified, dynamic and complicated [13].

As the urban agglomeration with the strongest comprehensive strength in China, the Yangtze River Delta urban agglomeration has become an important highland of China’s tourism development by virtue of its unique location conditions and advantages of resource and environment. According to the National Economic and Social Development Statistical Bulletin of relevant provinces, the total tourism revenue of the Yangtze River Delta urban agglomeration in 2019 is 3.91 trillion, accounting for 59% of the total national tourism revenue. However, while making considerable economic benefits, it also faces a series of problems such as environmental pollution, ecological destruction, resource waste. As the pioneering demonstration area of high-quality development in China, the Yangtze River Delta urban agglomeration has always been ahead of other regions in terms of economic and social development, ecological environmental protection, and technological innovation ability, and plays a leading role as a pioneer model.

Hence, studying the tourism eco-efficiency and its spatial network structure of the Yangtze River Delta Urban Agglomeration, and clarifying the position and interaction of different cities in the spatial association network can help us to understand the spatial association characteristics of tourism eco-efficiency in the Yangtze River Delta region as a whole, so as to put forward targeted suggestions to improve the tourism eco-efficiency in various cities. It can also provide new ideas for promoting the coordination and unification of economic, social and environmental benefits, and achieving green and balanced development. Moreover, it also provides theoretical guidance for the effective formulation of regional coordinated development policies. On the other hand, China is also an important tourist destination and tourist source country in the world. At present, its tourism industry is in a critical period of comprehensive optimization and upgrading. As the sixth largest urban agglomeration in the world, the Yangtze River Delta urban agglomeration is rising rapidly. Paying attention to the spatial-temporal evolution characteristics and spatial association structure of its tourism eco-efficiency can not only provide experience for the sustainable development of tourism in other regions of China, but also provide Chinese case and Chinese experience for the industrial upgrading and the regional integration coordinated development of urban agglomerations in other countries, which has important international exemplary significance and reference value.

In recent years, the sustainable development of tourism industry has attracted great attention from domestic and foreign scholars. In foreign countries, Gössling discussed environmental pollution caused by energy consumption in the tourism industry from the perspective of developing countries, and proposed countermeasures [14]. Santos-Lacueva et al. used policy analysis method to assess the vulnerability of coastal tourism destinations to climate change [15]. Paramati et al. verified the relationship between tourism investment, tourism revenue and carbon dioxide emission in EU countries through a panel data model [16]. Sustainable tourism behavior is also one of the current research hotspots [17,18,19]. Yilmaz et al. took Turkey as a case to study the impact of eco-label on sustainable tourism behavior of accommodation industry [20]. Taking Madrid as an example, Chamarro et al. assessed the differences between residents and visitors in their attitudes and behaviours towards sustainable tourism [21]. Predicated on the tri-component attitude model, Agyeiwaah et al. conducted an in-depth study on the sustainable behavior of backpackers [22]. Grilli et al. studied the prospective tourist preferences for sustainable tourism development in small island developing states through a mixed method combining potential factor analysis and choice experiment models [23]. At the same time, scholars have also studied the sustainable development of different types of tourism [24,25]. Based on IOA-NRM approach, Lin explored the sustainable development strategies of industrial tourism in Taiwan [26]. Choi et al. planned an eco-tourism system for estuary wetlands using a resilience principle-based systems thinking approach [27]. Some scholars have discussed the connotation and mechanism of sustainable tourism development from a theoretical perspective. Pan et al. summarized the relationship between tourism and sustainable development from an interdisciplinary perspective, and put forward implementation strategies for sustainable tourism from various aspects [28]. Based on recent changes in understanding of development and contemporary sustainable development approaches, Sharpley re-examined the theoretical relationship between tourism and sustainable development [29].

In China, Liu et al. [30] and Su et al. [31] respectively conducted research on the sustainable development of tourism in urban and rural areas. The environmental impact caused by tourism has also received widespread attention [32,33]. Zeng et al. found that there is an environmental Kuznets curve in China’s tourism industry, and tourism development has a significant spatial spillover effect on air pollution [34]. Liu et al. established a long-range energy alternatives planning system-Tourist model to observe and predict tourism greenhouse gas peaks in China from 2017 to 2040 [35]. Scholars have calculated the tourism carbon emission at the national [36], regional [37], provincial [38], and scenic scale [39]. Jin et al. took Jiangsu Province as an example to analyze the carbon emission flow of self-driving tourism and its determinants [40]. Luo et al. explored the driving factors of China’s tourism carbon emissions using index and structural decomposition methods [41]. Tang et al. analyzed the spatial-temporal evolution of China’s tourism carbon emissions from 2000 to 2015 and discussed the decoupling effects between tourism-related carbon emissions and tourism economy with the decoupling index [42]. In addition, domestic scholars have also made fruitful achievements in tourism ecological footprint and carbon footprint [43,44,45], and tourism green productivity [46,47].

Many scholars have studied the eco-efficiency of tourism industry from multiple perspectives. Chen et al. assessed the regional eco-efficiency and tourism economy development level of 31 provinces in China, and used a spatial panel econometric model to discuss the impact of tourism economy development on regional eco-efficiency and its spatial effect [48]. Pan et al. analyzed the evolution trend of China’s tourism carbon emission efficiency from 2007 to 2017, and explored the coupling coordination relationship among tourism carbon emissions, economic development and regional innovation [49]. Liu et al. measured the tourism eco-efficiency of 53 coastal cities in China, and used the Tobit model to explore the factors affecting the tourism eco-efficiency of coastal areas [50]. Based on the panel data from 2007 to 2013, Zha et al. calculated and analyzed the development efficiency and dynamic fluctuation of low-carbon tourism economy in Hubei Province [51]. Sun et al. comparatively analyzed the spatial-temporal evolution characteristics and internal differences of the tourism eco-efficiency in China’s three major urban agglomerations, and used a panel regression model to further explore the internal and external factors affecting the tourism eco-efficiency [52]. Besides, studies on the eco-efficiency of small-scale tourist destinations have gradually emerged [53,54]. Taking Huangshan national park as an example, Peng et al. analyzed the evolution characteristics and influencing factors of the eco-efficiency in tourism destination, and put forward corresponding suggestions [55].

In general, the existing studies about tourism eco-efficiency mainly focus on its spatial pattern evolution and influencing factors and rarely analyze the spatial association and the overall network dynamic characteristics. With the increasing attention of scholars to the spatial elements, research on the spatial network structure of tourism eco-efficiency has also been gradually carried out, and the current relevant researches are mainly concentrated in China. Liu and Song measured the green innovation efficiency of tourism industry in 30 provinces of China, and used the social network analysis method to study its spatial network structure and its formation mechanism [56]. Wang et al. explored the spatial network structure and its effect of China’s tourism eco-efficiency with the aid of a modified gravity model and social network analysis [57]. However, the above studies mainly focus on the spatial association of tourism eco-efficiency at the provincial level. In fact, affected by the level of economic development, the structure of tourism industry and other factors, the relationship between the tourism eco-efficiency of cities in a region presents complex and diverse network structure characteristics. There is a relatively lack of literature on the spatial association characteristics and evolution trend of regional tourism eco-efficiency, especially the spatial synergy and its effect within specific urban agglomeration. In addition, traditional radial data envelopment analysis (DEA) model [50,58] or non-radial slack-based measure (SBM) model [59,60] are mostly used to measure the tourism eco-efficiency in most studies. Both of these two methods have some limitations, which often lead to deviations in the measurement results of efficiency value, thus affecting the scientificity and accuracy of the research conclusion.

This paper aims to explore the spatial interaction structure of tourism eco-efficiency and its effect in urban agglomerations. Taking the Yangtze River Delta urban agglomeration as the research object, we first construct an evaluation indicators system for urban tourism eco-efficiency and calculate the tourism eco-efficiency of 41 cities from 2008 to 2017. Then, the spatial-temporal pattern evolution characteristics of tourism eco-efficiency and the variation of differences within the urban agglomeration are analyzed. Furthermore, we use the modified gravity model to determine the spatial association matrix of tourism eco-efficiency. On this basis, the social network analysis method is used to explore the spatial network structure characteristics of tourism eco-efficiency in the Yangtze River Delta urban agglomeration and its influence on tourism eco-efficiency. Finally, according to the conclusions, we put forward targeted policy implications to improve the tourism eco-efficiency of the cities in the Yangtze River Delta urban agglomeration.

The innovations of this research are mainly manifested in three aspects: Firstly, a hybrid distance model called super-epsilon-based measure (EBM) model is used to measure the tourism eco-efficiency. This model considers both the radial proportion information and non-radial slack variables, which overcomes the shortcomings of traditional radial and non-radial models, and makes the measurement results of tourism eco-efficiency more accurate [61]. Secondly, we introduce the social network analysis method to study the spatial association of tourism eco-efficiency from the perspective of network, and analyze the relationship between the network structure characteristics and eco-efficiency in the Yangtze River Delta urban agglomeration. Moreover, we divide 41 cities in the region into four blocks, clarify the position and role of each city in the network, and explore the spatial synergy and spillover effects between the various blocks. Thirdly, existing related studies almost only focus on the spatial association of tourism eco-efficiency at the provincial level, which can only provide general theoretical guidance from the macro level. Therefore, this paper further refines the research scale of the spatial association structure to the prefecture level, and discusses the spatial interaction characteristics of tourism eco-efficiency in various cities, so as to make the research results more specific and obtain more targeted policy recommendations.

The rest of this paper is structured as follows: Section 2 introduces the methodology, including methods and data. Section 3 presents the measurement results of the tourism eco-efficiency. Section 4 analyzes the characteristics of spatial network structure of tourism eco-efficiency. Section 5 discusses the research results. The final section provides the conclusion and policy implications.

## 2. Methodology

### 2.1. Measurement of Tourism Eco-Efficiency

#### 2.1.1. Super-EBM Model Based on Undesired Output

At present, the traditional DEA model and the SBM model are usually used to measure the eco-efficiency of tourism industry, which are relatively widely used. But in fact, these two methods have certain drawbacks. The traditional DEA model only considers the same proportional change information of input-output elements, and ignores the slack of input and output, which makes the measured eco-efficiency higher than the actual value [60]. The SBM model contains non-radial slack variables and adds the undesired output, but it also loses the original proportion information of the efficiency frontier projection value, resulting in a relatively low calculation result of the model. To solve this problem, Tone and Tsutsui proposed a hybrid EBM model compatible with radial proportions and non-radial slack in 2010 [62]. This model not only considers the radial proportion information between the actual input-output value and the target value, but also takes the influence of non-radial slack variable into account, which can more accurately measure the efficiency value of decision-making unit (DMU). The linear program of the unoriented EBM model based on undesired output are as follows:

(1) $$\gamma^{*}\;=\;\min\frac{\;\theta \;-\;\varepsilon_{x}\sum_{i=1}^{m}\frac{w_{i}^{-}s_{i}^{-}}{x_{i0}}}{\varphi \;+\;\varepsilon_{y}\sum_{r=1}^{s}\frac{w_{r}^{+}s_{r}^{+}}{y_{r0}}+\;\varepsilon_{u}\sum_{p=1}^{q}\frac{w_{p}^{-}s_{p}^{-}}{u_{p0}}}$$

(2) $$s.t.\;\sum_{j=1}^{n}x_{ij}\lambda_{j}{\;+\;s}_{i}^{-}{\;=\;\theta x}_{i0},\;i=\;1,2,\ldots ,\;m$$

(3) $$\overset{n}{\underset{j=1}{\sum }}y_{rj}\lambda_{j}-s_{r}^{+}{\;=\;\varphi y}_{r0},\;r=\;1,2,\ldots ,\;s$$

(4) $$\;\sum_{j=1}^{n}u_{pj}\lambda_{j}\;{+\;s}_{p}^{-}{\;=\;\varphi u}_{p0},\;p\;=\;1,2,\ldots ,\;q$$

(5) $$\lambda_{j}\;\geq {\;0,\;s}_{i}^{-}{,\;s}_{r\;}^{+}{,\;s}_{p}^{-}\;\geq \;0$$

where $\gamma^{*}$ represents for the eco-efficiency value; $\lambda_{j}$ denotes the linear combination coefficient of DMU*j*; $x_{ij},{\;y}_{rj},\;u_{pj}$ indicate the *i*-th input, *r*-th desirable output, and *p*-th undesired output of DMU*j*, respectively; *n, m, s, q* represent the number of DMUs, inputs, desirable outputs, and undesired outputs, respectively; $s_{i}^{-}$, $s_{r}^{+},\;s_{p}^{-}$ stand for the input slack, desirable output slack and undesired output slack, respectively; $w_{i}^{-}$, $w_{r}^{+}$, $w_{p}^{-}$ are the relative importance of each input, desirable output, and undesired output, respectively; θ refers to the radial planning parameter; $\varepsilon_{x\;},\;\varepsilon_{y\;},\;\varepsilon_{u}$ represent the non-radial weight of input, desirable output, and undesired output respectively, and the value range is [0, 1].

Since the calculation result of the EBM model still does not exceed 1, it is impossible to further compare multiple DMUs that are in the production frontier at the same time. Therefore, we use the method of Andersen and Petersen to improve the ordinary EBM model into a super-efficiency model with the best efficiency value greater than 1 [63], and measure the tourism eco-efficiency by using the Super-EBM model based on undesired output. When $\gamma^{*}\geq 1$, the eco-efficiency of the DMU is effective; when it is less than 1, the eco-efficiency of input-output elements is considered to be in an invalid state. Eco-efficiency is also called comprehensive efficiency (CE), which is composed of pure technical efficiency (PTE) and scale efficiency (SE). Pure technical efficiency can reflect the efficiency level of tourism in technology and management. Scale efficiency reflects the influence of the scale effect. When SE≥1, it means that the current production scale is in the best state. Comprehensive efficiency = pure technical efficiency × scale efficiency.

#### 2.1.2. Indicators System Construction

The essence of tourism eco-efficiency is to pay attention to the cost of resources and environment while developing tourism economy. In other words, tourism eco-efficiency is the efficiency of tourism development that takes resource consumption and environmental impact into account. In view of this, based on the available data, this paper constructs an evaluation indicators system of urban tourism eco-efficiency as shown in Table 1. The indicators system includes three parts: input, desired output and undesired output:

*Input indicators*: In the sense of traditional economics, labor, capital and land are the most basic production factors. Especially for tourism, a typical tertiary industry, labor [64] and capital [65] are essential input elements for industrial development. Wang et al. believe that the capital investment in the process of tourism production can indirectly reflect the condition of land investment in the tourism industry [66]. In addition, the large amount of energy consumption generated by tourism transportation, accommodation, catering and other departments cannot be ignored [67]. Therefore, labor, capital and energy consumption are selected as input indicators for measuring the eco-efficiency of tourism industry. Considering the availability of city-level data, this paper selects the number of employees in the tertiary industry to represent labor input, and the fixed asset investment in the tertiary industry to represent capital input. The fixed asset investment is reduced to the constant price based on 2008 according to the fixed asset investment price index over the years. Drawing on the research of Zhang et al. [68], tourism energy consumption is separated from the tertiary industry energy consumption by the tourism development coefficient (the ratio of tourism income to the tertiary industry’s GDP). The specific calculation formula is as follows:

(6) $$E_{t}\;=\;\underset{ij}{\sum }E_{ij,t}{\;\times \;\beta }_{j}{\;\times \;R}_{t}$$

where $E_{t}$ refers to the energy consumption of tourism in year *t*; *i* represents the *i*-th sector related to tourism in the tertiary industry; *j* represents the *j*-th energy type; $E_{ij,t}$ denotes the *j*-th energy terminal consumption of sector *i* in year *t*. $\beta_{j}$ indicates the standard coal conversion coefficient of energy *j*, referring to “General principles for calculation of the comprehensive energy consumption (GB/T2589-2008)”. $R_{t}$ is the tourism development coefficient in year *t*. The energy consumption of prefecture-level cities is obtained through the proportion of tourism revenue of each city in the whole province.

*Desirable output indicator*: The economic benefits generated by tourism industry can most intuitively reflect the tourism development of a city. Tourism income is generally considered to be a suitable indicator of desired output [49,69,70]. Therefore, the total tourism revenue is selected as the desired output, which consists of domestic and inbound tourism revenue. The inbound tourism revenue is converted by the exchange rate of US dollar to RMB over the years and reduced to the constant price in 2008 according to the consumer price index (CPI).

*Undesired output indicators*: Regarding the choice of undesired output indicators, scholars hold different opinions. Cheng et al. took the three wastes of tourism, namely garbage, sewage, and waste gas emissions as undesired output indicators [71]. However, Wang et al. [72] and Zha et al. [51] used tourism CO_2_ emissions to characterize the undesired output of tourism industry. Due to the particularity of tourism industry, it produces relatively less sulfur dioxide and industrial smoke, while the carbon emissions produced by the burning of fossil energy in tourism activities are relatively high. Therefore, based on the previous research experience, this paper selects tourism CO_2_ emission, tourism wastewater discharge, and tourism solid waste discharge to characterize the environmental impact caused by tourism, and uses entropy method to reduce dimensionality, as the undesired output of eco-efficiency measurement. Since there is no relevant statistical index for tourism environmental pollution, the tourism wastewater discharge is separated from the total wastewater discharge by the proportion of tourism income in GDP. The solid waste discharge of tourism industry is separated from the domestic waste clearance volume through tourism development coefficient. Tourism carbon emission is closely related to tourism energy consumption, which is the main source of tourism anthropogenic carbon emission [73]. Therefore, this paper calculates the tourism CO_2_ emission by energy consumption referring to the IPCC greenhouse gas emission algorithm and the method of Zhang et al. [68]. The formula is as follows:

(7) $${\mathrm{TC}}_{t}\;=\;\underset{ij}{\sum }E_{ij,t}{\;\times \;R}_{t}{\;\times \;V}_{j}{\;\times \;\mathrm{CEF}}_{j\;}{\times \;\mathrm{COF}}_{j\;}\times \;\frac{44}{12}$$

where ${\mathrm{TC}}_{t}$ stands for tourism CO_2_ emissions in year *t*; $V_{j}$ represents the average low calorific value of energy *j*; ${\mathrm{CEF}}_{j}$ and ${\mathrm{COF}}_{j}$ represent the carbon content per unit calorific value and the carbon oxidation rate of energy *j* respectively. In particular, the thermal carbon emissions are calculated based on the carbon content per unit calorific value of raw coal, and electricity carbon emissions are calculated according to the regional power grid baseline emission factor published by the China’s National Development and Reform Commission over the years.

### 2.2. Modified Gravity Model and Spatial Association Network

The construction of spatial association network is the basis of the application of social network theory and method. Gravity model and VAR model are the two most commonly used methods to determine spatial association network in current research [74]. The VAR model focuses on the hysteresis of variables, while the gravity model is more suitable for the measurement of gravity between regions. Zipf introduced gravity model to the study of urban spatial interaction for the first time, and proposed the corresponding formula [75]. Since then, gravity model has been widely used in various fields such as migration, trade, tourism, and transportation [76,77,78,79]. Drawing on the research of Wang et al. [57], this paper uses the modified gravity model to measure the spatial association intensity of tourism eco-efficiency in the Yangtze River Delta urban agglomeration. The formulae are as follows:

(8) $$F_{ab}=k_{ab}\times \;\frac{E_{a}\times E_{b}}{{\left(\frac{d_{ab}}{r_{a-}r_{b}}\right)}^{2}},\;k_{ab}=\frac{R_{a}}{R_{a}+R_{b}}$$

where *a* and *b* respectively denote city *a* and city *b*; $F_{ab}$ refers to the strength of the association pointing from city *a* to city *b*; $E_{a},\;\;E_{b}$ respectively represent the tourism eco-efficiency of city *a* and *b*; $R_{a}$, $R_{b}$ are the total tourism revenue of city *a* and *b* respectively; $k_{ab}$ is the contribution rate of city *a* to $F_{ab}$; $r_{a}$ and $r_{b}$ stand for the per capita tourism income of city *a* and *b* respectively; $d_{ab}$ indicates the spherical distance between two cities. The spatial association matrix of tourism eco-efficiency in the Yangtze River Delta urban agglomeration is obtained by the above formula. Then it is converted into a binary matrix with the mean value of each row as the threshold [80,81]. If the value is greater than the mean value, it is assigned as 1, indicating that there is a spatial connection between the two cities. If it is less than the mean value, it is assigned as 0. In this case, it is considered that there is no spatial connection. The spatial binary matrix of tourism eco-efficiency is the data basis of spatial network structure analysis.

### 2.3. Social Network Analysis(SNA)

Social network analysis (SNA) is a structural method based on the interactive research of various parts in the system, using the form of network to analyze its relationship mode and characteristics. SNA transforms attribute data into relational data through quantitative indicators. It not only reveals the characteristics and changing trend of the overall network, but also reflects the role played by individuals in the network and their relationship with other members. SNA explores the spatial association of a region as a whole, breaking away from the limitation of “adjacency” of traditional spatial measurement methods [82]. It provides algebraic analysis and visual expression of social relations [83], and has become a popular interdisciplinary academic method [84]. At present, the social network analysis method has been widely used in many fields such as tourism flow [85,86], energy consumption [87,88], environmental protection [89,90]. In this paper, the tourism eco-efficiency network is abstracted as a social network. A “node” in the network represents a city in the urban agglomeration, and the “line” between the nodes represents the spatial correlation between cities in terms of tourism eco-efficiency. The spatial association network of tourism eco-efficiency in urban agglomeration is thus constructed. We use SNA to explore the spatial network structure and evolution trend of the tourism eco-efficiency in the Yangtze River Delta urban agglomeration from three aspects: overall network characteristics, network centrality and block model analysis.

#### 2.3.1. Overall Network Characteristics

In research of spatial association network, four indicators of network association, network density, network hierarchy and network efficiency are usually used to describe the characteristics of the overall network structure [91]. Network association refers to the sum of the number of relations between each node city in the entire network. Network density is the ratio of the network association to the theoretical maximum number of relations, reflecting the closeness of the connections among nodes in the network. The greater the network density, the greater the influence of the network on each node. The network hierarchy reflects the asymmetric accessibility in the network. The higher the hierarchy, the more prominent the hierarchical structure of the network. Network efficiency is generally referred to the connection efficiency between nodes in spatial association network. The lower the network efficiency is, the more redundant lines in the network, namely the more overflow channels, so the higher the network stability is.

#### 2.3.2. Network Centrality

Network centrality is used to analyze the power and status of each node in the network. Degree centrality, betweenness centrality and closeness centrality are the three most commonly used metrics [92]. Degree centrality represents the degree of association between a node and other nodes in the network, and reflects the degree to which a node is located at the center of the network. It is divided into out-degree and in-degree in digraph. Out-degree represents the node’s ability to influence others, and in-degree represents the degree to which the node is affected by others. Betweenness centrality is used to measure the extent to which a node is in the “middle” of other nodes, reflecting the degree to which the node controls resources in the network. The greater the betweenness centrality is, the greater the power of the node to control other nodes, and vice versa. Closeness centrality reflects the ability of a node not to be “controlled” by others. If a node is closer to other nodes, its actions are not dependent on others, and the closeness centrality is higher.

#### 2.3.3. Block Model Analysis

Block model analysis was first proposed by Boorman and White [93]. It divides the nodes in the network into different blocks through clustering method, and then analyzes the role and interaction of various blocks in the spatial association network. Referring to Wasserman and Faust’s evaluation method [94], this paper divides the spatial association network of tourism eco-efficiency in the Yangtze River Delta urban agglomeration into four blocks, namely “net benefit block”, “net spillover block”, “two-way spillover block” and “agent block”, so as to explore the spatial relationship of tourism eco-efficiency between and within the blocks. The “net benefit block” receives relationships from its own members and other blocks, but the block receives significantly more relationships from the outside than it sends to the outside. On the contrary, the “net spillover block” sends far more relations to the outside than receives from the outside. The “two-way spillover block” not only sends relations to other blocks, but also receives relations from other blocks, but has more relations among its internal members. The “agent block” receives and sends out more external relations, and it has more external contacts and less internal contacts.

### 2.4. Research Area and Data Sources

**Figure 1:**
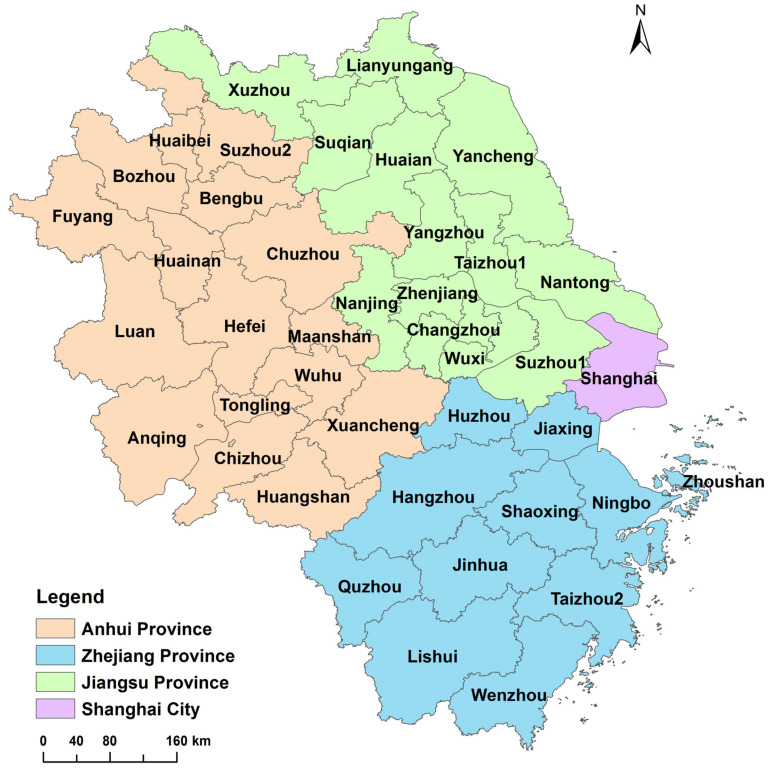
Location of the study area.

According to the “Outline of the Yangtze River Delta Regional Integration Development Plan” issued by the State Council of China in December 2019, the planning scope of the Yangtze River Delta region is extended to Jiangsu, Zhejiang, Shanghai and Anhui (as shown in Figure 1).

Since 2008, with the rapid development of transportation and information technology as well as the improvement of people’s living standards, the demand of domestic residents for leisure tourism has gradually expanded. At the same time, the success of the 2008 Beijing Olympic Games has brought huge development opportunities for China’s inbound tourism. As a result, China’s tourism industry developed rapidly and made great achievements during this period. In view of the fact that some of the data involved in this study have not been released in time by the relevant national authorities, the latest data are up to 2017. Therefore, this paper selects 2008 to 2017 as the research period to analyze the panel data of 41 cities in the Yangtze River Delta urban agglomeration. Since Chaohu City in Anhui Province was split in 2011, to ensure the consistency of statistical results, only 16 prefecture-level cities in Anhui Province are considered here.

The data used in this paper are from the “China City Statistical Yearbook”, “China Energy Statistical Yearbook”, “China City Construction Statistical Yearbook”, each city’s statistical yearbook, and the national economic and social development statistical bulletin over the years. The missing values of individual years are supplemented by average growth rate method or average value method. In addition, the spherical distance between the cities is obtained by using Python to calculate the distance between the two government stations. The calculation formulas of related indicators in the overall network analysis and centrality analysis are in the literature [91]. For the sake of distinction, Suzhou1 and Suzhou2 are used to denote Suzhou City in Jiangsu Province and Suzhou City in Anhui Province respectively. Taizhou1 and Taizhou2 refer to Taizhou City in Jiangsu Province and Taizhou City in Zhejiang Province, respectively.

## 3. Measurement of Tourism Eco-Efficiency in the Yangtze River Delta Urban Agglomeration

### 3.1. Spatial-Temporal Evolutionary Characteristics of Tourism Eco-Efficiency

This paper uses the Super-EBM model to measure the tourism eco-efficiency of 41 cities in the Yangtze River Delta urban agglomeration by using MAX DEA 8.0 software, and analyzes its spatial distribution pattern and evolution trend.

From the provincial level, the tourism eco-efficiency of the Yangtze River Delta region is ranked as follows: Jiangsu > Zhejiang > Anhui > Shanghai, and the gap between provinces is significant. As shown in Table 2, the average value of tourism eco-efficiency in the Yangtze River Delta from 2008 to 2017 was 0.742, among which the average value of Jiangsu reached 0.920, close to the effective state. The second is Zhejiang, which had a relatively high level of efficiency with average value exceeded 0.85. The tourism eco-efficiency in Anhui and Shanghai were lower than the overall average of the Yangtze River Delta, only 0.604 and 0.587 respectively. On the whole, the eco-efficiency of tourism industry in the Yangtze River Delta from 2008 to 2017 showed a fluctuating downward trend. Compared with that of ten years ago, the eco-efficiency value of each province decreased to varying degrees in 2017. Specifically speaking, the overall tourism eco-efficiency in the region declined from 2008 to 2009 and reached its peak in 2010. After falling to a trough in 2011, it experienced a small increase process and began to decline year by year since 2013.

**Figure 2:**
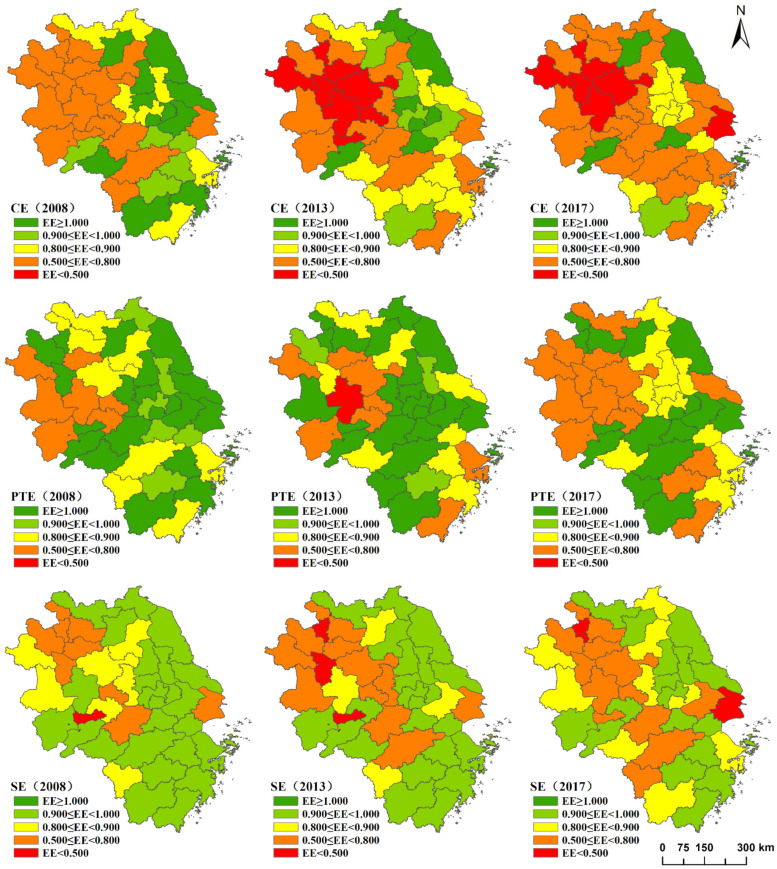
Spatial and temporal pattern evolution of tourism eco-efficiency in the Yangtze River Delta urban agglomeration. In 2008, 2013 and 2017.

The above analysis at the provincial level can only reflect the average level of the province’s tourism eco-efficiency. In fact, there are also large differences between different prefecture-level cities in the same province, so it is necessary to further analyze the eco-efficiency value of each city. This paper selects 2008, 2013 and 2017 as time sections and uses ArcGIS 10.2 software for visual expression. Figure 2 clearly shows the spatial pattern and evolution of the comprehensive efficiency, pure technical efficiency, and scale efficiency in the Yangtze River Delta urban agglomeration.

(1) As shown in Figure 2, the comprehensive efficiency of all cities in the Yangtze River Delta in 2008 was not less than 0.5. High-efficiency areas were mainly distributed in the southeast coast of Jiangsu, south-central Zhejiang, and scattered in southwest Anhui. Among them, Wuxi, Suzhou1, Yancheng, Nantong, Yangzhou, Zhenjiang, Suqian in Jiangsu Province, Zhoushan, Taizhou2, Lishui in Zhejiang Province and Huangshan in Anhui Province had a comprehensive efficiency greater than 1, which reached the best eco-efficiency. In other words, the input and output of tourism industry in these cities were in an effective state. However, the comprehensive efficiency of Shanghai and most areas in Anhui Province is relatively low, below 0.8. From 2008 to 2017, the comprehensive efficiency of most cities in the region gradually declined. In 2013, there were only 6 cities with the best efficiency, and only 4 cities remained in 2017. In 2017, the comprehensive efficiency value of Shanghai, Hefei, Bengbu, Huainan, Fuyang, Huaibei and Chuzhou was already less than 0.5. In the whole Yangtze River Delta urban agglomeration, only Yancheng, Huzhou, Quzhou and Chizhou have higher comprehensive efficiency than ten years ago.

(2) In terms of pure technical efficiency, high-efficiency areas were concentrated in central and southern Jiangsu, southern Anhui and central Zhejiang in 2008, while low-efficiency areas were mainly distributed in western Anhui. In contrast to comprehensive efficiency, the pure technical efficiency of Shanghai was always greater than 1 in 2008–2017, which means that Shanghai has a high level of low-carbon technology and management, maintaining an optimal state. From 2008 to 2013, the pure technical efficiency of coastal cities such as Nantong, Ningbo, Wenzhou and inland Hefei and Huangshan decreased significantly. Among them, the efficiency value of Hefei dropped below 0.5. At the same time, Changzhou, Lianyungang, Hangzhou, Huzhou and Chuzhou increased significantly. From 2013 to 2017, only Hefei and Lianyungang increased their pure technical efficiency, while other cities remained basically unchanged or declined.

(3) With regard to scale efficiency, as a whole, all cities in the Yangtze River Delta region did not reach the best scale efficiency from 2008 to 2017. In 2008, the distribution of scale efficiency in the Yangtze River Delta was relatively even. Large areas of Jiangsu and Zhejiang, and southwest Anhui belonged to the high efficiency concentration area, while Shanghai and Xuancheng, Bozhou, Maanshan in Anhui were low. Tongling is the only city with scale efficiency less than 0.5 in the region. From 2008 to 2013, the efficiency value of central and northern Anhui dropped significantly. In 2013, low-efficiency cities with efficiency value below 0.5 increased to Tongling, Huainan, and Huaibei. From 2013 to 2017, the number of high-efficiency cities gradually decreased. The scale efficiency of central and western Anhui increased to some extent, while the eastern cities of Shanghai, Suzhou1, Wuxi, Ningbo, and Lianyungang declined significantly. Among them, the scale efficiency of Shanghai fell below 0.5 in 2017.

### 3.2. Differences within the Yangtze River Delta Urban Agglomeration

**Figure 3:**
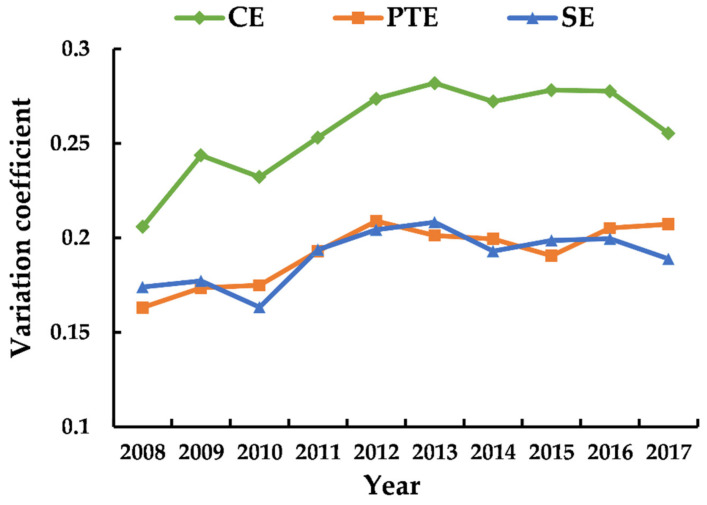
Variation coefficient of tourism eco-efficiency in the Yangtze River Delta urban agglomeration.

By calculating the variation coefficient of the tourism eco-efficiency in the Yangtze River Delta urban agglomeration in the past ten years, this paper explores the variation degree of tourism eco-efficiency within the Yangtze River Delta. The result is shown in Figure 3. In general, the variation coefficient of the comprehensive efficiency, pure technical efficiency and scale efficiency in the Yangtze River Delta urban agglomeration increased to varying degrees from 2008 to 2017. It can be seen that compared with ten years ago, the gap of eco-efficiency within the urban agglomeration has widened. Since 2008, the variation coefficient of the comprehensive efficiency in the Yangtze River Delta showed a trend of fluctuating upward. After reaching the peak in 2013, it began to fluctuate downward, which means that the tourism eco-efficiency gap within the region first increased and then decreased. The variation coefficient of pure technical efficiency experienced a “N” type change process of up-down-up, rising from 2008 to 2012, gradually decreasing from 2013 to 2015, and then rising steadily. As for the scale efficiency, the variation trend of the variation coefficient is similar to that of the comprehensive efficiency, but the change range is slightly smaller than it. It can be inferred that the change of tourism eco-efficiency depends on the change of scale efficiency to a great extent, and scale efficiency is the key factor to determine the eco-efficiency.

## 4. Characteristics of Spatial Network Structure for Tourism Eco-Efficiency

### 4.1. Analysis of Overall Network Structure

**Figure 4:**
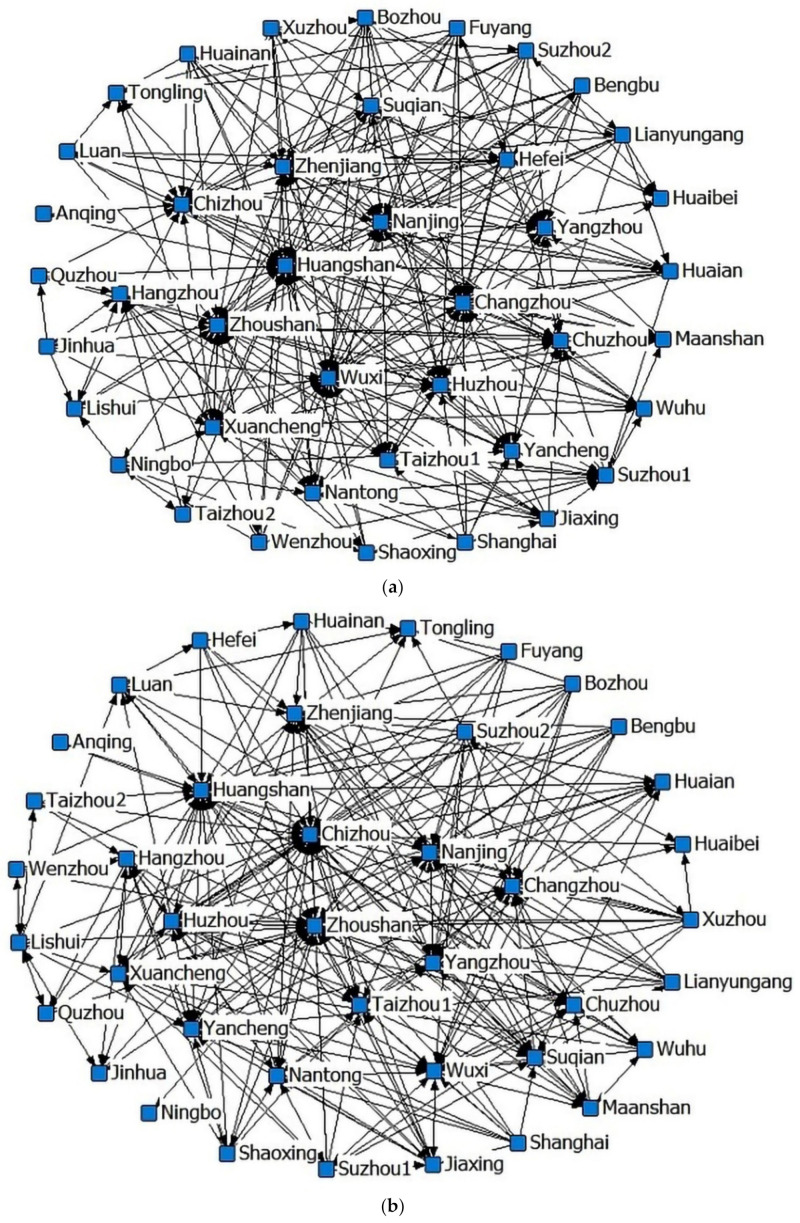

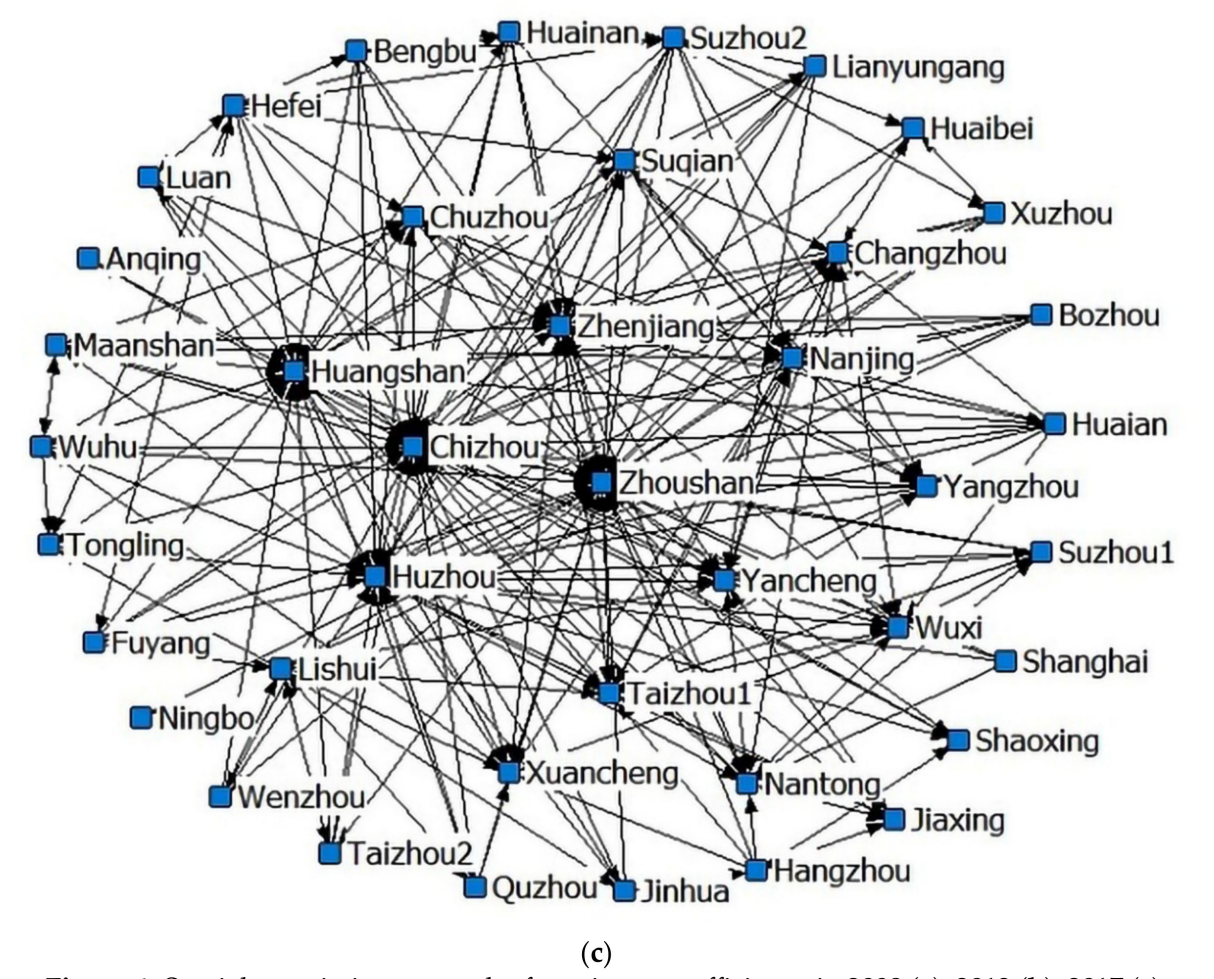
Spatial association network of tourism eco-efficiency in 2008 (**a**), 2013 (**b**), 2017 (**c**).

Based on the cross-sectional data of 2008, 2013 and 2017, the spatial association matrix of tourism eco-efficiency is obtained according to formula (8) and transformed into a binary matrix. Using Netdraw tool in UCINET 6.0 software, the spatial network structure of tourism eco-efficiency in the Yangtze River Delta urban agglomeration is drawn as shown in Figure 4. In the figure, each node represents each city in the Yangtze River Delta. The connection between the nodes indicates that there is a spatial connection, and the arrow points to the overflow direction. As can be seen from Figure 4, from 2008 to 2017, the spatial association network of tourism eco-efficiency in the Yangtze River Delta had become increasingly sparse, and the central cities in the network were constantly changing. While a few cities such as Huangshan, Huzhou and Zhoushan have always maintained the central position of the network.

**Figure 5:**
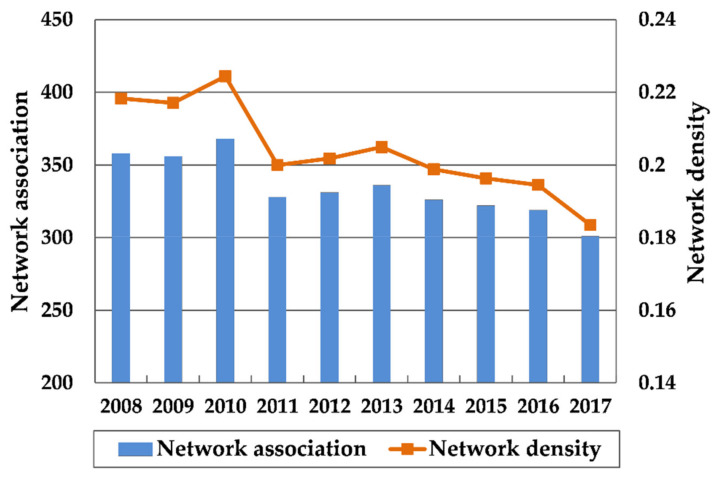
Network association and network density.

**Figure 6:**
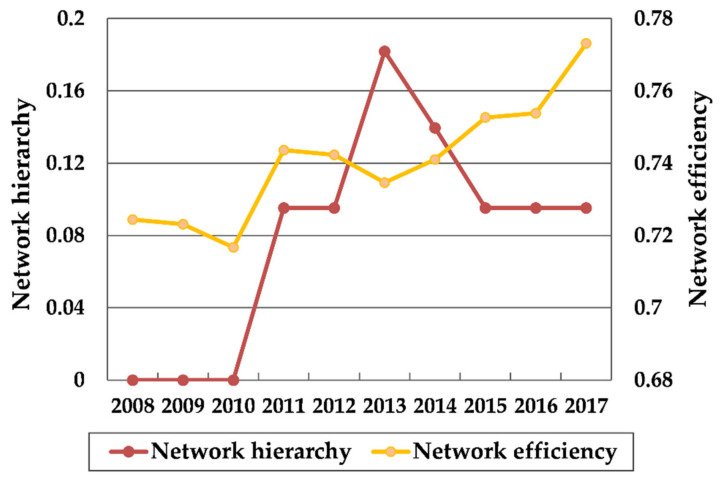
Network hierarchy and network efficiency.

The network association number, network density, network hierarchy and network efficiency of the Yangtze River Delta urban agglomeration were calculated by UCINET 6.0 software, and the change trend from 2008 to 2017 was visualized (Figure 5 and Figure 6). In order to explore the correlation between the tourism eco-efficiency and the characteristics of the overall network structure, the mean value of regional tourism eco-efficiency was analyzed by Pearson correlation with network density, network hierarchy and network efficiency through SPSS software. The results are shown in Table 3.

(1) Network association and network density

As shown in Figure 5, the spatial network association of tourism eco-efficiency in the Yangtze River Delta shows a downward trend. In 2008, there were 358 association relations in the region, but only 301 remained in 2017. During this period, it reached a small peak in 2010 with the largest number of association relations at 368. Correspondingly, the overall network density also dropped from 0.218 in 2008 to 0.184 in 2017, and the peak in 2010 was only 0.224. It means that the spatial network of tourism eco-efficiency in the Yangtze River Delta urban agglomeration was not closely connected, and it tended to be loose gradually. As can be seen from Table 3, there is a significant positive correlation between tourism eco-efficiency and network density in the Yangtze River Delta, and the correlation coefficient is as high as 0.904. That is to say, the greater the spatial network association is, the higher the regional tourism eco-efficiency is, and vice versa. To a large extent, the weakening of the network connection strength of the Yangtze River Delta urban agglomeration led to the decrease of the overall regional tourism eco-efficiency.

(2) Network hierarchy and network efficiency

It can be seen from Figure 6 that the network hierarchy remained at 0 from 2008 to 2010, indicating that during this period of time, a strict hierarchical structure had not been established in the spatial association network of tourism eco-efficiency in the Yangtze River Delta region, and the green development of each city was in a relatively balanced state. In the next three years, the network hierarchy increased significantly, which means that the asymmetric accessibility in the network increased gradually. After reaching a peak of 0.182 in 2013, the network hierarchy began to gradually decline, and stabilized at around 0.095 in 2015 2017. The overall network efficiency showed a fluctuating upward trend, from 0.724 in 2008 to 0.773 in 2017, which indicates that the number of network association in the Yangtze River Delta was decreasing. That is to say, the spatial spillover channels were decreasing and the network stability was gradually decreasing. As can be seen from Table 3, the correlation coefficients between tourism eco-efficiency and network efficiency, network hierarchy are −0.898 and −0.643 respectively, both of which have passed the significance test of 5%, showing negative correlation. In other words, the tourism eco-efficiency in the Yangtze River Delta urban agglomeration decreases with the increase of network efficiency and network hierarchy.

In 2010, the “Regional Plan for the Yangtze River Delta Region” approved by the State Council of China greatly promoted the cooperation and exchanges in various fields such as industrial development, infrastructure, resource utilization, and environmental protection in the Yangtze River Delta region. The coordination of regional development enhanced, so the network density of the tourism eco-efficiency in the Yangtze River Delta urban agglomeration also experienced a short-term increase in 2010. However, due to the large differences in the location conditions, economic development level, tourism resource endowment, and environmental regulation intensity of each city, tourism investment and income still tended to be concentrated in a small number of cities with superior congenital conditions driven by market mechanisms. As a result, the network association of eco-efficiency gradually decreased. It can also be seen from Figure 3 that since 2011, the gap of tourism eco-efficiency within the Yangtze River Delta had been expanding, which was closely related to the decline in the overall cohesion of the network.

### 4.2. Network Centrality Analysis

Using UCINET 6.0, this paper calculates the degree centrality, betweenness centrality and closeness centrality of the tourism eco-efficiency in the Yangtze River Delta, so as to analyze the centrality of each city in the spatial association network. As shown in Table 4, the mean value of the degree centrality of the tourism eco-efficiency in the Yangtze River Delta urban agglomeration in 2017 was 26.463. The top four cities are Zhoushan, Chizhou, Huzhou and Huangshan, indicating that they have extremely close links with other cities in the region in terms of eco-efficiency. There is a common feature among these cities, that is, they are all tourist cities known for beautiful scenery and pleasant environment. They have rich and high-quality eco-tourism resources, and their tourism development level is relatively high. The tourism eco-efficiency of these cities is in the forefront of the Yangtze River Delta. In terms of in-degree, 14 cities including Zhoushan, Chizhou, Huzhou, and Huangshan are above the average. These cities are susceptible to the promotion of the eco-efficiency of other cities and have strong attraction to various resource elements in the region. Therefore, most of them belong to high-efficiency cities with eco-efficiency value greater than 0.8. Regarding out-degree, the top five cities are Huangshan, Huzhou, Hefei, Zhenjiang and Suqian. They have a strong radiation effect on the outside world in terms of eco-efficiency and can effectively promote the tourism eco-efficiency of other cities.

It can be seen from Table 4 that the average betweenness centrality of the Yangtze River Delta urban agglomeration in 2017 was 3.099, and the polarization among cities in the region was obvious. The betweenness centrality of Huangshan was the largest (23.525), which means that Huangshan was at the core of the spatial association network. It is the “bridge” and “link” to connect the cities in the Yangtze River Delta, and has a strong ability to control the information and resources in the region. On the contrary, the betweenness centrality of Xuzhou, Bozhou and Shanghai was 0, indicating that they did not act as any “intermediary” between cities, and were relatively marginal in the eco-efficiency spatial network. The reasons for this result are different. Xuzhou and Bozhou are restricted by location conditions and tourism development level so that their influence in the network is relatively weak. As China’s financial center, the tourism development mode of Shanghai is mainly exhibition tourism, theme park tourism and modern urban tourism. The eco-tourism resources are relatively weak, and the utilization rate is low. It was always in the stage of diminishing returns to scale with large input scale but low output. In the aspect of eco-tourism, there was also a lack of contact with other cities, which has led to the relatively low eco-efficiency of its tourism industry.

Closeness centrality reflects the degree to which each city in the network is not dominated by others. According to the measurement results in Table 4, there are 8 cities in the Yangtze River Delta urban agglomeration whose closeness centrality exceeds the mean value of 58.146. From largest to smallest, they are Zhoushan, Chizhou, Huzhou, Huangshan, Zhenjiang, Yancheng, Taizhou1 and Suzhou2. Among them, the closeness centrality of Zhoushan is much higher than that of other cities, reaching 93.023. This means that these cities are relatively close to other cities in the eco-efficiency network, and can quickly establish connections with other cities, acting as the central actor in the spatial network. The closeness centrality of cities such as Anqing, Ningbo and Tongling is relatively low. They have not established close ecological connection with other cities, and are in a relatively passive position in the spatial association network, which has led to the low eco-efficiency of tourism in these cities. This may be related to the relatively remote location.

### 4.3. Block Model Analysis

In order to explore the clustering characteristics of the spatial association network of the Yangtze River Delta urban agglomeration, this paper uses the convergence of iterated correlation method (CONCOR) in UCINET 6.0 software for block model analysis based on the data of tourism eco-efficiency in 2017. Taking 2.0 as the maximum segmentation depth and 0.2 as the convergence standard, the 41 cities in the Yangtze River Delta urban agglomeration are divided into four blocks according to their locations. The results are shown in Table 5. Block 1 consists of five cities: Nanjing, Wuxi, Yangzhou, Changzhou and Zhenjiang, all located in southern Jiangsu. This block receives 46 relations from the outside and sends 38 relations to the outside. The actual internal relationship ratio is 11.63%, which is greater than the expected internal relationship ratio of 10.00%. Therefore, it is judged that block 1 is the “two-way spillover block”. Block 2 contains six cities of Huzhou, Zhoushan, Hangzhou, Lishui, Chizhou and Huangshan, which belong to Zhejiang and Anhui province respectively. For the second block, the number of receiving relations from the outside is much greater than the number of relations sent to the outside by itself. Thus, we judge it to be the “net benefit block”, which is consistent with the higher eco-efficiency of tourism in these cities. Block 3 includes 18 cities such as Xuzhou, Hefei, Lianyungang, Fuyang, and Jinhua. This block has a total of 104 relations sent to the outside world, far exceeding the number of 31 relations it receives from the outside. Obviously, its net spillover effect is significant and it belongs to a “net spillover block”. Block 4 is composed of 11 cities, including Suzhou1, Shaoxing, Taizhou1, Nantong, etc. The number of relations received and sent by this block to the outside reach 54 and 61 respectively. In addition, the expected relationship ratio within the block is greater than the actual internal relationship ratio. Hence, it is considered that block 4 belongs to the “agent block” and plays an important role of “intermediary” and “bridge” in the spatial association network.

In order to further explore the relationship among the four blocks, the density matrix of the spatial association network is constructed. Taking the overall network density of 0.184 as the threshold, the value of greater than 0.184 is assigned to 1, and the value of less than 0.184 is assigned to 0. In this way, the multi-value density matrix is converted into an image matrix. According to Table 6 and Table 7, it can be seen that only block 1 has a relatively close internal connection, the internal connection of the other three blocks is weak. The total number of associations between blocks is 256, far exceeding the number of internal associations of 45. There are different degrees of connection between various blocks. Among them, there is a two-way overflow relationship between block 1 and block 4, block 2 and block 3. It can be seen that the high-efficiency cities in block 2 are largely dependent on the spillover effect of block 3because block 2 receives as many as 71 relations from block 3. Generally speaking, although the various blocks in the network have obvious clustering characteristics, their internal spatial relationships are not close. On the contrary, the synergy and spillover effects among the four blocks are more significant, and there is a spatial polarization centered on a few cities with superior resource endowments.

## 5. Discussion

Based on the calculation of the tourism eco-efficiency in the Yangtze River Delta urban agglomeration from 2008 to 2017, this paper explores the spatial-temporal evolution characteristics of the tourism eco-efficiency and the differences within the urban agglomeration. Furthermore, we use the modified gravity model and SNA to conduct in-depth research on the structural characteristics of the spatial association network of tourism eco-efficiency, and try to explore the effect of the spatial network structure on the tourism eco-efficiency. Although our research takes the Yangtze River Delta urban agglomeration as an example, which is a developed coastal urban agglomeration in China, this method can also be applied to explore the spatial association of tourism eco-efficiency in other urban agglomerations in China and even in the world. It provides a new research paradigm for the study of green tourism development in urban agglomerations.

First of all, different from previous studies focusing on the tourism eco-efficiency and its spatial correlation at the provincial level, this paper takes the Yangtze River Delta urban agglomeration as an example to explore the spatial-temporal evolution trend of regional tourism eco-efficiency, spatial synergy characteristics and its influence effects on the tourism eco-efficiency. The research scale of tourism eco-efficiency is further refined to the prefecture level. Besides, the hybrid Super-EBM model including both radial and non-radial distance functions is used to measure the eco-efficiency, which makes up for the shortcomings of the traditional radial DEA model and non-radial SBM model to a certain extent, and makes the calculation results of eco-efficiency more real and accurate. The results indicate that the overall eco-efficiency of tourism in the Yangtze River Delta urban agglomeration from 2008 to 2017 showed a fluctuating downward trend. In 2017, the tourism eco-efficiency of each province in the region decreased to varying degrees compared with ten years ago, which is consistent with the research conclusion drawn by Sun et al. [52]. At the same time, the research of Wang et al. shows that the overall tourism eco-efficiency of China’s 31 provinces also showed a downward trend from 1997 to 2016 [70]. It can be seen that the rapid growth of tourism industry has failed to compensate for the pollution and damage to the ecological environment, which is a common problem throughout the country. From 2008 to 2017, the average eco-efficiency of tourism industry in the Yangtze River Delta region is 0.742, and there is a significant gap between provinces. Jiangsu Province and Zhejiang Province are higher than the regional average level with the average eco-efficiency reaching 0.920 and 0.857 respectively. The input and output of tourism industry in Jiangsu Province are basically close to the effective state. While the eco-efficiency of Anhui Province and Shanghai City is relatively low, only 0.604 and 0.587 respectively. There is still a certain gap between the current level and the frontier of efficiency, which needs to be further improved.

Secondly, the variation coefficient is used to explore the variation of the tourism eco-efficiency differences within the Yangtze River Delta urban agglomeration. The results show that, the variation coefficient of tourism comprehensive efficiency, pure technical efficiency and scale efficiency in the Yangtze River Delta urban agglomeration in 2017 has increased compared with ten years ago. From 2008 to 2013, the variation coefficient of the comprehensive efficiency in the Yangtze River Delta showed a fluctuating upward trend. After reaching the peak in 2013, it began to decline. It can be seen that the comprehensive efficiency gap within the Yangtze River Delta urban agglomeration showed a trend of first increasing and then decreasing. Compared with ten years ago, the gap within the region has expanded. The change of comprehensive efficiency depends to a large extent on the change of scale efficiency, which is consistent with the conclusion drawn by Peng et al. on the tourism eco-efficiency of Huangshan National Park [55].

Thirdly, through the method of social network analysis, this paper explores the structural characteristics of spatial association network of tourism eco-efficiency in the Yangtze River Delta urban agglomeration. From the overall network structure characteristics, the spatial connection of the tourism eco-efficiency in the Yangtze River Delta is not close. In recent years, the network association has been decreasing and the network structure has gradually become loose. There is a significant positive correlation between the regional tourism eco-efficiency and network density, and the weakening of network association strength has largely led to the decrease of the overall tourism eco-efficiency in the Yangtze River Delta. From 2008 to 2017, the hierarchical structure in the network has grown from scratch, and the spatial barriers in ecological connection of cities have gradually formed. The network efficiency shows a fluctuating upward trend, and the stability of spatial association network gradually declines. The regional tourism eco-efficiency decreases with the increase of network hierarchy and network efficiency. This conclusion is also confirmed by Wang et al.’s research on the spatial association network of provincial tourism eco-efficiency [57]. This means that the quality of the spatial network structure affects the overall regional tourism eco-efficiency to a certain extent.

From the perspective of network centrality, the degree centrality of Zhoushan, Huzhou, Huangshan and Chizhou is much higher than the average level of the Yangtze River Delta urban agglomeration. Because of it, they have strong attraction to various resource elements in the region, so that their tourism eco-efficiency is also high. The top five cities in terms of out-degree are Huangshan, Huzhou, Hefei, Zhenjiang and Suqian, which have a strong radiation effect on the outside. The city with the largest betweenness centrality is Huangshan, which occupies a core position in the spatial association network and plays the role of “bridge” and “link” in the spatial network of tourism eco-efficiency. The betweenness centrality of Xuzhou, Bozhou and Shanghai is 0, indicating that they are relatively marginal in the network. The closeness centrality of Zhoushan, Chizhou, Huzhou, Huangshan, Zhenjiang, Yancheng, Taizhou and Suzhou is much higher than the regional average. They are easy to establish ecological connection with other cities and play the role of central actor in the network. While the closeness centrality of cities such as Anqing, Ningbo and Tongling is relatively low, and their tourism eco-efficiency is also lower than the regional average. Previous studies have also shown that the improvement of network centrality can significantly promote the improvement of tourism eco-efficiency [57]. Therefore, cities on the edge of the network must attempt to improve their central position in the network to promote the tourism eco-efficiency.

According to the results of block model analysis, the 41 cities in the Yangtze River Delta urban agglomeration are divided into four blocks. The block composed of Nanjing, Wuxi, Yangzhou, Changzhou, and Zhenjiang belongs to the “two-way spillover block” in the spatial association network of tourism eco-efficiency. The block consisting of Huzhou, Zhoushan, Hangzhou, Lishui, Chizhou, and Huangshan plays the role of “net benefit”, and the number of external relations it receives is far greater than the number of external relations issued by itself. Correspondingly, the tourism eco-efficiency of the cities in this block is relatively high. The block composed of 18 cities including Xuzhou, Hefei, Lianyungang, Fuyang and Jinhua plays a “net spillover” role, which has obvious net spillover effect. The block composed of 11 cities such as Suzhou1, Shaoxing, Taizhou1 and Nantong plays the role of “agent” in the spatial association network, it receives and emits a large number of relations to the outside. Generally speaking, the clustering characteristics of the blocks in the network are obvious, but the spatial association within each block is relatively loose. There is a significant spillover effect and synergy between the blocks, as well as a spatial polarization phenomenon centered on a few cities. This conclusion is similar to the research result of Liu et al. on the green innovation efficiency of China’s tourism industry [56].

It should be noted that this study still has some limitations. Firstly, tourism eco-efficiency is a comprehensive concept involving many factors such as economy, society and environment. Due to the difficulty in obtaining data of prefecture-level cities, the selection of indicators for measuring tourism eco-efficiency may not be comprehensive, and the design of the indicators system needs to be further optimized. Secondly, if the relationship between tourism eco-efficiency and spatial network structure can be quantitatively analyzed using a model method, the dynamic mechanism affecting tourism eco-efficiency will be more accurately reflected, so that we can put forward more targeted suggestions and measures. These issues are research directions worthy of in-depth discussion in the future.

## 6. Conclusions and Policy Implications

An excellent ecological environment is the prerequisite and foundation for the sustainable development of tourism. Against the background of increasingly serious ecological environment problems, how to improve the quality of ecological environment to ensure the sustainable development of tourism industry has become a problem and test that the whole society needs to face together. As the link between tourism economy, resources and environment, tourism eco-efficiency takes full consideration of resource consumption and environmental impact caused by tourism activities while paying attention to tourism development. It is an important tool to evaluate the sustainable development of regional tourism.

Based on the panel data from 2008 to 2017, this paper constructs an indicators system for evaluating the urban tourism eco-efficiency. Using the Super-EBM model based on the undesired output, this paper measures the tourism eco-efficiency of 41 prefecture level cities in the Yangtze River Delta urban agglomeration, and deeply analyzes the evolution characteristics of its spatial-temporal patterns and changes in internal differences. In addition, this paper uses the modified gravity model to construct the spatial association matrix, and further discusses the spatial network structure of tourism eco-efficiency and its evolution trend in the Yangtze River Delta from three aspects of overall characteristics, individual characteristics and block characteristics of the spatial association network. The conclusions are as follows:

(1) From 2008 to 2017, the average eco-efficiency of tourism industry in the Yangtze River Delta urban agglomeration is 0.742. In this decade, the tourism eco-efficiency generally showed a fluctuating downward trend. The gap between provinces is significant. Specifically, the tourism eco-efficiency of Jiangsu and Zhejiang is relatively high, while Anhui and Shanghai are lower than the regional average and there is a large room for improvement. The variation coefficient of the tourism eco-efficiency in the Yangtze River Delta experienced a process of increasing first and then decreasing. Compared with ten years ago, the variation coefficient of tourism eco-efficiency has increased in 2017. In other words, the internal gap of the tourism eco-efficiency within the Yangtze River Delta urban agglomeration has gradually widened.

(2) The network density of tourism eco-efficiency decreased from 0.218 in 2008 to 0.184 in 2017, while the network hierarchy and network efficiency have increased. It can be seen that the spatial network of the tourism eco-efficiency in the Yangtze River Delta urban agglomeration is not closely connected, and tends to be loose gradually. In addition, the hierarchical structure of spatial association network has gradually increased, and network stability has also declined. The research shows that tourism eco-efficiency is positively correlated with network density, but negatively correlated with network hierarchy and network efficiency.

(3) According to the analysis of network centrality, the spatial association network of the tourism eco-efficiency in the Yangtze River Delta urban agglomeration has a typical “core-periphery” structure. High-efficiency cities such as Huangshan, Zhoushan, Chizhou, and Huzhou are always located in the center of the network, maintaining close ties with other cities. However, Cities such as Xuzhou, Bozhou, Anqing, Ningbo and Tongling are relatively marginal in the network, and their tourism eco-efficiency is relatively low.

(4) The 41 cities in the Yangtze River Delta urban agglomeration can be divided into four blocks: “two-way spillover”, “net benefit”, “net spillover” and “agent”. The “two-way spillover block” is composed of five cities in central and southern Jiangsu: Nanjing, Wuxi, Yangzhou, Changzhou, and Zhenjiang. The “net benefit block” consists of high-efficiency cities of Huzhou, Zhoushan, Hangzhou, Lishui, Chizhou and Huangshan. The “net spillover block” is made up of 18 cities including Xuzhou, Hefei, Lianyungang and Fuyang. Eleven cities, such as Suzhou1, Shaoxing, Taizhou1, and Nantong, constitute the “agent block”. There is a significant spillover effect and synergy between the blocks, as well as a spatial polarization phenomenon centered on a few cities, while the spatial association within each block is relatively loose.

Based on the above conclusions, we put forward the following policy implications:

Firstly, local governments must deepen institutional reform, establish and improve the cooperation and exchange mechanisms in various aspects, so as to strengthen regional internal ties through the means of macro-control. It is found that the tourism eco-efficiency in the Yangtze River Delta urban agglomeration presents a spatial association network structure. The tourism eco-efficiency of each city not only depends on its own tourism development, but also is affected by other cities around it. However, the current green development of tourism in the Yangtze River Delta urban agglomeration has problems such as loose regional connection and large internal gap, which affect the improvement of the overall tourism eco-efficiency in the Yangtze River Delta region. Therefore, local governments need to focus on strengthening cooperation and exchanges in tourism, environmental protection, technology and other aspects, and issue corresponding policies and measures. In terms of tourism development, we suggest integrating regional resource advantages, accurately positioning the tourism image of the Yangtze River Delta, and creating a regional tourism brand with distinctive features. At the same time, strengthen cooperation in the development and sale of tourism products. For example, launch excellent cross-regional tourist routes and implement a joint ticket system of scenic spots, etc. Moreover, local governments should work together to strengthen the publicity and promotion of the overall tourism brand, enhance the popularity of regional tourism, thereby sharing the tourism market and achieving a win-win cooperation in regional tourism development. In the aspect of environmental protection, local governments should jointly formulate laws and regulations on regional pollution prevention and control, and unify the standards of environmental quality and pollutant discharge. It is necessary to build a regional ecological environment monitoring network and environmental information sharing platform to share real-time information on environmental quality, pollution emissions, and pollution control technology. Meanwhile, it must clarify the supervision and management responsibilities of departments at all levels, and adhere to the collaborative governance of environmental problems such as air pollution, wastewater, solid waste, and carbon emissions. Superior government should reduce the cost of element flow, break down the cooperation barriers caused by the administrative system, so as to promote the efficient circulation and optimal allocation of resource elements in the region. For instance, a series of policies such as tax incentives, loan subsidies and product R&D subsidies are adopted to guide capital flow to areas with low eco-efficiency of tourism.

In addition, each city should take corresponding measures to promote the improvement of tourism eco-efficiency according to its position and role in the spatial association network. High-efficiency cities such as Huangshan, Zhoushan, Huzhou, and Chizhou are always at the core of the network, and they are closely related to other cities in terms of tourism eco-efficiency. However, these cities belong to the “net benefit block”, which has a strong attraction to the resource elements of surrounding cities. As a result, while improving their own tourism eco-efficiency, it also weakens the ability of other cities to develop green tourism. These high-efficiency cities located in the center of the network should make full use of the spatial connection with other cities to play their radiation effect and leading role. Further strengthen their function as bridges and ties in the region, and drive the improvement of the tourism eco-efficiency in low-efficiency cities. Xuzhou, Hefei, Lianyungang, Fuyang and other cities in the “net spillover block” are mostly located at the edge of the network. Due to the loss of their own resource elements, their tourism eco-efficiency has also been affected. These cities should take the initiative to take measures to strengthen the links with other cities in all aspects, such as improving transportation infrastructure to advance the traffic accessibility. At the same time, it is also significant to introduce high technology and perfect the talent introduction policy. They are also supposed to draw lessons from high-efficiency cities on energy-saving and emission-reduction paths and pollution control experience, and organically combine them with tourism industry to reduce environmental damage and pollution caused by tourism. The “two-way spillover block” is composed of Nanjing, Wuxi, Yangzhou, Changzhou and Zhenjiang, which are five cities in central and southern Jiangsu. These cities belong to the more developed cities in the region, and their tourism eco-efficiency is relatively high. They should be based on their strong economic foundation and give full play to their advantages in technology and talents. Increase investment in technological innovation, promote the application of new generation information technology in the tourism industry, so as to use innovation to drive the sustainable development of tourism. Cities in the “agent block” such as Suzhou1, Shaoxing, Taizhou1 and Nantong are closely connected with the outside world. By optimizing industrial structure and increasing energy efficiency, they can enhance their intermediary role while improving their own tourism eco-efficiency, so as to promote the green and balanced development of the tourism industry in the Yangtze River Delta urban agglomeration.

## Figures and Tables

**Table 1:** Input–output indicators system

Indicator Type	Indicator Name	Primary
Input indicators	Labor input Capital input Energy input	Number of employees in the tertiary industry Fixed asset investment in the tertiary industry Tourism energy consumption
Desirable output indicator	Total tourism economy	Total tourism revenue
Undesirable output indicators	Tourism environmental pollution	Tourism CO_2_ emission Tourism wastewater discharge Tourism solid waste discharge

**Table 2:** Provincial tourism eco-efficiency in the Yangtze River Delta urban agglomeration from 2008 to 2017.

Province	2008	2009	2010	2011	2012	2013	2014	2015	2016	2017	Average
Jiangsu	0.938	0.939	0.927	0.925	0.928	0.923	0.938	0.939	0.930	0.812	0.920
Zhejiang	0.905	0.867	0.871	0.830	0.861	0.865	0.851	0.847	0.839	0.828	0.857
Shanghai	0.623	0.563	0.658	0.602	0.676	0.636	0.590	0.525	0.514	0.486	0.587
Anhui	0.682	0.640	0.642	0.609	0.579	0.570	0.585	0.580	0.575	0.582	0.604
Mean value	0.787	0.752	0.775	0.742	0.761	0.749	0.741	0.722	0.715	0.677	0.742

**Table 3:** Correlation between tourism eco-efficiency and overall network structure characteristics.

Explanatory Variables	Network Density	Network Efficiency	Network Hierarchy
Pearson correlation coefficient	0.904 **	−0.898 **	−0.643 *
*p*-value	0.000	0.000	0.045

Note: ** and * indicate significant at the level of 1% and 5% (bilateral) respectively.

**Table 4:** Centrality analysis of spatial association network of tourism eco-efficiency in the Yangtze River Delta.

City	Degree Centrality			Betweenness Centrality	Closeness Centrality	City	Degree Centrality			Betweenness Centrality	Closeness Centrality
	In-Degree	Out-Degree	Centrality				In-Degree	Out-Degree	Centrality		
Nanjing	10	9	32.500	2.319	57.971	Zhoushan	37	9	92.500	16.830	93.023
Wuxi	8	9	27.500	0.661	57.143	Taizhou2	2	5	12.500	1.124	53.333
Xuzhou	1	8	20.000	0.000	55.556	Lishui	6	9	27.500	1.272	57.143
Changzhou	8	8	25.000	2.738	57.143	Shanghai	0	4	10.000	0.000	56.338
Suzhou1	3	7	17.500	0.079	54.795	Hefei	4	11	27.500	8.314	57.971
Nantong	9	9	27.500	3.182	57.971	Wuhu	4	8	20.000	0.176	55.556
Lianyungang	1	8	20.000	1.734	55.556	Bengbu	2	8	20.000	0.256	55.556
Huaian	2	9	22.500	0.329	56.338	Huainan	3	7	20.000	2.546	55.556
Yancheng	13	9	35.000	4.196	60.606	Maanshan	7	8	20.000	1.686	55.556
Yangzhou	8	7	27.500	0.248	57.971	Huaibei	3	3	12.500	0.064	53.333
Zhenjiang	17	10	45.000	5.315	62.500	Tongling	6	2	15.000	0.293	51.282
Taizhou1	12	9	32.500	4.116	59.701	Anqing	2	2	5.000	0.107	47.059
Suqian	5	10	27.500	5.109	57.971	Huangshan	25	14	65.000	23.525	74.074
Hangzhou	3	7	20.000	0.307	54.054	Fuyang	1	7	17.500	0.138	54.795
Ningbo	1	1	2.500	0.075	48.780	Suzhou2	4	10	30.000	3.392	58.824
Wenzhou	2	5	12.500	1.124	53.333	Chuzhou	9	9	27.500	1.641	57.971
Jiaxing	4	7	17.500	1.164	54.795	Luan	3	6	15.000	7.442	54.054
Huzhou	27	13	72.500	10.570	76.923	Xuancheng	11	6	27.500	2.715	57.971
Shaoxing	3	5	12.500	1.390	53.333	Chizhou	31	5	77.500	9.273	80.000
Jinhua	2	6	15.000	1.105	54.054	Bozhou	0	6	15.000	0.000	54.054
Quzhou	2	6	15.000	0.511	54.054	Mean value	7.341	7.341	26.463	3.099	58.146

**Table 5:** Spillover effect of the spatial association blocks of tourism eco-efficiency in the Yangtze River Delta.

Block	Number of Cities	Reception		Spillover		Expected Internal Relationship Ratio (%)	Actual Internal Relationship Ratio (%)	Block Attributes
		Internal	External	Internal	External			
Block 1	5	5	46	5	38	10.00	11.63	two-way spillover block
Block 2	6	4	125	4	53	12.50	7.02	net benefit block
Block 3	18	27	31	27	104	42.50	20.61	net spillover block
Block 4	12	9	54	9	61	27.50	12.86	agent block

Notes: Expected internal relationship ratio = (number of cities within the block-1)/(number of cities in the network-1); Actual internal relationship ratio = number of internal relationships of blocks/total number of spillover relationships of blocks.

**Table 6:** Density matrix and image matrix of the spatial correlation block of tourism eco-efficiency in the Yangtze River Delta.

Block	Density Matrix				Image Matrix			
	Block 1	Block 2	Block 3	Block 4	Block 1	Block 2	Block 3	Block 4
Block 1	0.250	0.333	0.111	0.300	1	1	0	1
Block 2	0.067	0.133	0.185	0.431	0	0	1	1
Block 3	0.311	0.657	0.088	0.023	1	1	0	0
Block 4	0.267	0.611	0.005	0.068	1	1	0	0

**Table 7:** Inter-block linkages of tourism eco-efficiency in the Yangtze River Delta.

Relationship between Blocks	Block 1	Block 2	Block 3	Block 4
Block 1	5	10	10	18
Block 2	2	4	20	31
Block 3	28	71	27	5
Block 4	16	44	1	9

## Data Availability

The data presented in this study are available on request from the corresponding author.
